# Safety of options to ″Boost″ (enhancing insulin infusion rates) and ″Ease-off″ (reducing insulin infusion rates) in CamAPS FX Hybrid Closed-Loop system: a real-world analysis

**DOI:** 10.1089/dia.2024.0298

**Published:** 2024-09-09

**Authors:** Chloë Royston, Simon Bergford, Peter Calhoun, Judy Sibayan, Yue Ruan, Charlotte Boughton, Malgorzata E Wilinska, Roman Hovorka

**Affiliations:** 1 Institute of Metabolic Science-Metabolic Research Laboratories, University of Cambridge, Cambridge, UK; 2 Jaeb Center for Health Research, Tampa, Florida, USA; 3 Shenzhen Institute of Advanced Technology, Chinese Academy of Sciences, Shenzhen, China

**Keywords:** Type 1 diabetes, hybrid closed-loop, CamAPS FX, Boost, Ease-off

## Abstract

The usage and safety of the Boost and Ease-off features in the CamAPS FX hybrid closed-loop system was analysed in a retrospective analysis of real-world data from 7,464 users over a 12-month period. Boost was used more frequently than Ease-off, but for a shorter duration per use. Mean starting glucose was above range for Boost (229±51 mg/dL), and within range for Ease-off (114±29 mg/dL). Time spent below 70 mg/dL was low during Boost periods [median (IQR) 0.0% (0.0, 0.5%)], and lower than during no Boost periods [2.1% (1.2, 3.4%)], while time spent above 180 mg/dL was lower during Ease-off periods (15±14%) compared to no Ease-off periods (25±12%). There were no episodes of severe hypoglycaemia or DKA attributed to Boost or Ease-off use. Boost and Ease-off allow users to engage safely with CamAPS FX to manage their glucose levels during periods of more-than-usual and less-than-usual insulin needs.

## Introduction

Hybrid closed-loop insulin delivery systems have become the standard of clinical care to manage type 1 diabetes (1). Hybrid closed-loop systems, through their glucose-responsive insulin delivery, improve glycaemic outcomes, increasing time in range without increasing the risk of hypoglycaemia in people with type 1 diabetes. (2)

The CamAPS FX hybrid closed-loop system comprising of the interoperable CamAPS FX app, a compatible continuous glucose monitor (CGM) and a compatible insulin pump, has been shown to be safe and effective in randomised clinical trials (3–6), and in real-world analyses (7) across a wide range of ages (≥2 years old) including during pregnancy. Boost and Ease-off are unique features of the CamAPS FX app, which allow enhanced customisability for users, so that the risk of hypoglycaemia can be reduced during periods of increased insulin sensitivity such as exercise (Ease-off), and glucose control can be improved when insulin sensitivity is lower such as during intercurrent illness, stress or hormonal changes associated with menstrual cycles or puberty (Boost). (8)

It is important to evaluate the safety and usage of the Boost and Ease-off features to ensure that these features are utilised appropriately, Boost does not increase the risk of hypoglycaemia, and Ease-off does not increase the risk of hyperglycaemia. The objective of the present study was to analyse the usage and safety of Boost and Ease-off across all age groups using real-world data.

## Materials and methods

### Study design and study population

Twelve months of real-world data collected between 1 December 2022 and 30 November 2023 was retrospectively analysed. Users aged between 1 and 80 years old using CamAPS FX (control algorithm version 0.3.71; CamDiab, Cambridge, UK), Dexcom G6 (Dexcom, CA, USA) and YpsoPump (Ypsomed, Burgdorf, Switzerland), and who provided consent to share their data were included. Data were analysed for individuals who used closed-loop for a minimum of 8 weeks. The data-set includes users with varying duration of use of CamAPS FX.

### CamAPS FX closed-loop system

CamAPS FX is an interoperable hybrid closed-loop app compatible with the Dexcom G6 and FreeStyle Libre 3 (Abbott Diabetes Care, Alameda, CA, USA) continuous glucose monitors, and the YpsoPump, DANA Diabecare RS and DANA-I insulin pumps (Sooil, Seoul, Republic of Korea). The app, installed on a standard Android smartphone, uses an adaptive model predictive control algorithm for titrating insulin delivery. (9) The algorithm is initialised by entering the user’s weight and total daily insulin dose. Insulin sensitivity and active insulin time are automatically calculated and adjusted by the algorithm as necessary. The control algorithm is highly adaptive by adjusting total daily insulin requirements, diurnal variations, and insulin delivery around meals. The default glucose target is 104 mg/dL but this can be adjusted between 80 mg/dL and 200 mg/dL in segments of 30 minutes (10).

The CamAPS FX app has two features, Boost and Ease-off, providing enhanced customisability to the users at times of higher or lower insulin needs, respectively. During Boost use, the control algorithm increases the amount of insulin delivered by assuming higher insulin needs corresponding to approximately a 35% increase of the total daily dose (TDD) in a glucose-responsive manner; there is no change in glucose target. During Ease-off, the glucose target and insulin sensitivity are increased causing the control algorithm to deliver less insulin.

Users are advised that Boost can be used when insulin requirements are higher (e.g., during illness, growth surges, certain times of the menstrual cycle or times of unusual hyperglycaemia), while Ease-off can be used during periods when less insulin is needed and/or insulin sensitivity is higher (e.g., during exercise or times of increased hypoglycaemia risk). (11)

### Data analysis

Each sensor glucose reading was determined to be either in Boost mode, Ease-off mode, or neither mode. Glucose endpoints were calculated for each mode if the participant had ≥4 hours in the respective mode. Only data from periods with closed-loop mode on were included. All endpoints were calculated per participant giving equal weight to each user.

Analyses were conducted across all users, and across six pre-defined age groups: 1-6 years, 7-13 years, 14-17 years, 18-22 years, 23-65 years and ≥66 years old. Mean sensor glucose, time spent in range 70-180 mg/dL, time below 70 mg/dL, below 54 mg/dL, above 180 mg/dL and above 250 mg/dL were calculated for each population. Mean duration, frequency of use (number of times per week), and mean glucose on initialisation of each feature was calculated for periods when Boost was on and when Ease-off was on. Percentage of time with sensor glucose availability and closed-loop use while features were in use was also calculated. Episodes of severe hypoglycaemia or diabetic ketoacidosis (DKA) were collected through the post-market surveillance process which includes a wide range of data from multiple sources, from all 15 countries where CamAPS FX is available. Relevant data was inspected to determine whether Boost or Ease-off were used prior to or during these events.

Data analysis was completed using SAS software, version 9.4 (SAS Institute, Cary, NC, USA). Data is presented as mean ± standard deviation for normally distributed data or median (interquartile range) for non-normally distributed data.

## Results

A total of 7,464 users who met the inclusion criteria were included in the analysis. Mean age was 32±19 years. A median of 89 (58, 119) days of sensor glucose data per user was analysed. The median percentage of time with sensor glucose and closed-loop on while Boost or Ease-off were in use was 97% (93, 99%) and was consistent across all age groups between 95 and 98% (Table 1).

### Boost and Ease-off usage

**Figure 1:**
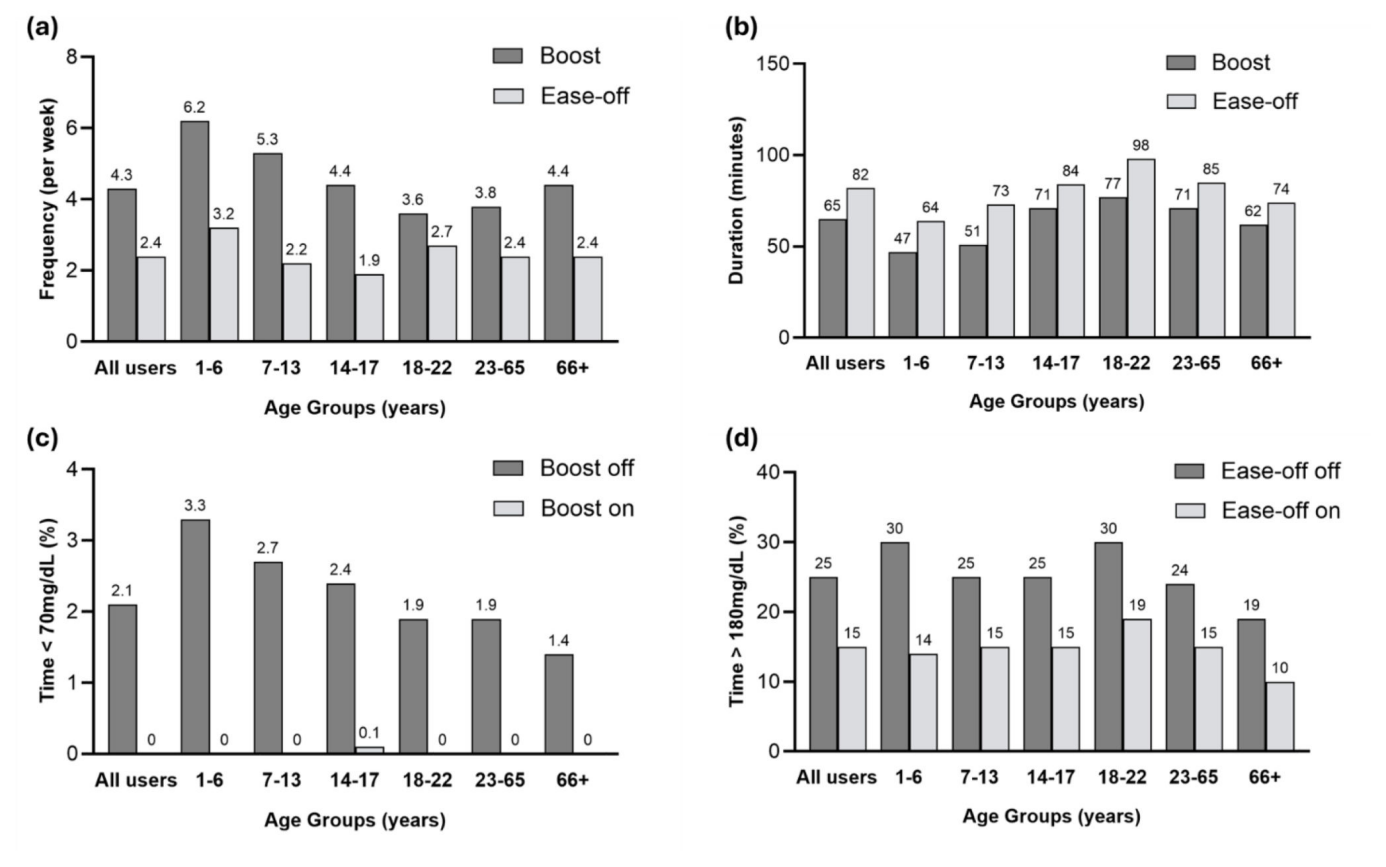
(a) Frequency of Boost and Ease-off use (b) Duration of each Boost and Ease-off period (c) Percentage of time below range when Boost is on compared to Boost off (d) Percentage of time above range when Ease-off is on compared to Ease-off off.

Endpoints stratified by age group are provided in Table 1 and 2. Boost was used more frequently than Ease-off at median 4.3 (1.8, 8.6) times per week compared to 2.4 (1.0, 5.4) times per week, but for a shorter duration of 65 (43, 98) minutes for Boost compared to 82 (56, 120) minutes for Ease-off (Figure 1). Boost was used the most frequently in very young children (1-6 years old) at 6.2 (3.1, 11.5) times per week, while young adults (18-22 years old) used it the least often at only 3.6 (1.4, 8.0) times per week. The duration of Boost was shortest for very young children at 47 (31, 68) minutes and longest for young adults at 77 (51, 112) minutes (Table 1). Similar patterns were observed with Ease-off, with its use most frequent in very young children at 3.2 (1.4, 8.5) times per week but for the shortest duration of 64 (42, 99) minutes. Young adults had the longest duration of Ease-off use at 98 (62, 143) minutes while teenagers (14-17 years old) used Ease-off the least often at a median of 1.9 (0.9, 4.2) times per week (Table 2). The frequency of use of Boost and Ease-off over the 24 hour period by age group (<18 years and ≥18 years) is shown in the Supplementary Appendix (Figure S1). The duration of Boost and Ease-off use by month for those participants starting closed-loop during the data collection period was sustained over time (Table S2).

### Sensor glucose endpoints

Time in range (70-180 mg/dL), above (>180 mg/dL) and below (<70 mg/dL) range are shown for periods when Boost was on versus off and when Ease-off was on versus off in Table 1 and 2. Mean glucose on initiating Boost was 229±51 mg/dL. There was very little time in hypoglycaemia during Boost periods; median time below 70 mg/dL was 0.0% (0.0, 0.5%) and time below 54 mg/dL was 0.0% (0.0, 0.0%) (Figure 2). During Boost, time in range was 28±22%, mean sensor glucose was 228±46 mg/dL, and time above range was 72±22%. Sensor glucose when Ease-off was initiated was 114±29 mg/dL. Time in range during Ease-off periods was 75±14%, mean sensor glucose was 127±27 mg/dL (Table 2), and time above 180 mg/dL was 15±14% (Figure 2). Median time above 250 mg/dL was low at 1.0% (0.0, 4.0%). Time below 70 mg/dL was 7.0% (2.9, 14.4%). In keeping with overall glycaemic trends in this age cohort, very young children had the highest time below range during Ease-off periods at 9.9% (3.6, 18.8%) (Table 2).

When neither Boost nor Ease-off feature was on, time in range was 73±12%, time below range was 2.1% (1.2, 3.4%), time above range was 25±12% and mean sensor glucose was 151±20 mg/dL.

Trends in glycaemic metrics by age categories were similar during Boost on, Ease-off on and when neither Boost nor Ease-off were on, with older adults (≥66 years old) having the lowest mean sensor glucose, highest time in range and lowest time above range, with minimal time below range. Very young children had lowest time in range, highest time above range and relatively high time below 70 mg/dL (Table 1 and Table 2).

### Boost and Ease-off usage and serious adverse events

One severe hypoglycaemia event occurred when Boost was on, however this occurred when Boost was initiated shortly after a manual bolus overcorrection. One DKA episode occurred when Ease-off was on which was attributed to an infusion set failure.

## Discussion

Our analysis shows that Boost and Ease-off are frequently used features of the CamAPS FX hybrid closed-loop app,suggesting acceptability, ease-of-use, and a desire for some engagement with the system by the user. While other commercially available hybrid closed-loop systems usually have a feature that directs the system to target higher glucose values or reduce insulin delivery (e.g. activity mode) (12–14), none of the other systems have a function such as Boost, which increases insulin delivery to deal with periods of increased insulin resistance or insulin requirements. Our analysis shows that Boost was used more frequently than Ease-off across all age groups.

Caregivers of very young children, aged 1-6 years used the two features the most frequently. This likely reflects the unique challenges faced by this population including unpredictable activity, erratic meal consumption and intense management required to mitigate these challenges (15). Young adults aged 18-22 years tended to use these features less frequently which may reflect their reduced overall engagement with the app and diabetes management (15). This age group often struggles to meet recommended glycaemic targets due to the additional challenges and competing priorities of young adulthood such as moving away from home support systems, and starting higher education or work (16).

Boost appears to have been used for its intended purpose with a mean starting glucose of 229 mg/dL when Boost is turned on. Consequently, time in range during Boost periods was lower due to higher time spent in hyperglycaemia and higher starting glucose. Importantly from a safety perspective, time below 70 mg/dL and 54 mg/dL were very low during Boost use overall with median 0.0%, including in very young children and older adults where the risks and consequences of hypoglycaemia are greatest. Our results are similar to those observed in a previous analysis of Boost using data from clinical trials (17).

Ease-off was initiated when glucose was within target range with mean starting glucose 114 mg/dL, suggesting that users may be using Ease-off proactively, prior to becoming hypoglycaemic, while Boost is used in a more reactive way once users are already experiencing hyperglycaemia. As would be expected for a feature designed to be used during periods of increased insulin sensitivity, time spent below 70 mg/dL was higher during Ease-off periods than when Ease-off was not used (median 7.0% vs. 2.1%). Importantly, the reduced insulin delivery during Ease-off did not lead to significant hyperglycaemia, as the mean time above 180 mg/dL was 15% and the median time above 250 mg/dL was 1%.

Strengths of the present study include the wide range of ages included (between 1 and 80 years), the use of real-world data, and the frequent use of Boost and Ease-off across all age groups allowing evaluation of its safety in the most vulnerable age groups, including very young and elderly. Limitations include the retrospective nature of the analyses which does not allow us to understand the reason why users initiated Boost/Ease-off or provide a comparator of glucose outcomes if Boost/Ease-off were not intiiated, and the fact that the only demographic data available was the year of birth.

In conclusion, user-initiated closed-loop features Boost and Ease-off are safe. These features are frequently used by users across all age groups and most frequently by caregivers of young children. Boost and Ease-off allow users to engage safely with CamAPS FX to manage their glucose levels at periods of more-than-usual and less-than-usual insulin needs.

## Supplementary Material

Supplementary Appendix

## Figures and Tables

**Table 1:** Sensor glucose outcomes during Boost, Ease-off and neither periods using real-world data.

	All users	Young children Age 1 − 6 years	Pre-pubertal Children Age 7 − 13 years	Teenagers Age 14 − 17 years	Young adults Age 18 − 22 years	Adults Age 23 − 65 years	Older Adults Age 66 years and older
**Number of participants**	7464	705	991	515	421	4590	242
**Age** (years)	32 ± 19	5 ± 1	10 ± 2	15 ± 1	20 ± 1	42 ± 11	71 ± 4
**Amount of CGM data per participant** (days)	89 (58, 119)	91 (58, 124)	90 (58, 124)	80 (54, 109)	77 (49, 106)	90 (61, 120)	95 (59, 122)
**CGM and closed-loop usage**							
Time using CGM and closed-loop (%)	97 (93, 99)	97 (94, 100)	96 (92, 99)	95 (89, 99)	95 (90, 99)	97 (93, 100)	98 (95, 100)
**Boost usage**							
Mean duration of Boost (min)	65 (43, 98)	47 (31, 68)	51 (32, 75)	71 (48, 101)	77 (51, 112)	71 (49, 105)	62 (40, 85)
Frequency of Boost use (per week)	4.3 (1.8, 8.6)	6.2 (3.1, 11.5)	5.3 (2.4, 9.4)	4.4 (1.9, 9.3)	3.6 (1.4, 8.0)	3.8 (1.6, 7.8)	4.4 (2.0, 8.6)
**Ease-off usage**							
Mean duration of Ease-off (min)	82 (56, 120)	64 (42, 99)	73 (50, 114)	84 (52, 130)	98 (62, 143)	85 (60, 125)	74 (53, 101)
Frequency of Ease-off use (per week)	2.4 (1.0, 5.4)	3.2 (1.4, 8.5)	2.2 (1.0, 5.1)	1.9 (0.9, 4.2)	2.7 (1.0, 5.6)	2.4 (1.0, 5.3)	2.4 (1.2, 5.0)
**Sensor glucose: Boost on**							
Glucose at start of Boost	229 ± 51	245 ± 51	246 ± 51	242 ± 58	246 ± 56	221 ± 47	209 ± 41
Mean glucose (mg/dL)	228 ± 46	248 ± 46	245 ± 47	234 ± 49	238 ± 46	220 ± 44	214 ± 39
Time in range (%)	28 ± 22	19 ± 18	21 ± 19	26 ± 20	25 ± 19	31 ± 23	31 ± 23
Time <70mg/dL (%)	0.0 (0.0, 0.5)	0.0 (0.0, 0.4)	0.0 (0.0, 0.4)	0.1 (0.0, 0.8)	0.0 (0.0, 0.5)	0.0 (0.0, 0.4)	0.0 (0.0, 0.1)
Time <54mg/dL (%)	0.00 (0.00, 0.00)	0.00 (0.00, 0.00)	0.00 (0.00, 0.00)	0.00 (0.00, 0.10)	0.00 (0.00, 0.00)	0.00 (0.00, 0.00)	0.00 (0.00, 0.00)
Time >180mg/dL (%)	72 ± 22	81 ± 18	79 ± 19	73 ± 21	75 ± 20	69 ± 23	68 ± 23
Time >250mg/dL (%)	31 (14, 54)	47 (27, 68)	46 (24, 67)	36 (18, 59)	41 (22, 60)	26 (12, 46)	20 (9, 38)
**Sensor glucose: Ease-off on**							
Glucose at start of Ease-off	114 ± 29	118 ± 32	113 ± 28	108 ± 33	115 ± 31	114 ± 28	109 ± 28
Mean glucose (mg/dL)	127 ± 27	124 ± 25	127 ± 26	125 ± 29	134 ± 32	127 ± 27	120 ± 25
Time in range 70 to 180mg/dL (%)	75 ± 14	74 ± 13	74 ± 13	71 ± 14	71 ± 15	75 ± 14	80 ± 12
Time <70mg/dL (%)	7.0 (2.9, 14.4)	8.9 (4.4, 16.7)	7.5 (3.4, 15.9)	9.9 (3.6, 18.8)	6.1 (2.5, 12.9)	6.5 (2.5, 13.1)	6.6 (2.7, 14.7)
Time <54mg/dL (%)	0.94 (0.00, 3.03)	1.74 (0.44, 4.04)	1.27 (0.17, 3.44)	1.84 (0.24, 5.44)	0.85 (0.00, 2.82)	0.76 (0.00, 2.57)	0.74 (0.00, 2.18)
Time >180mg/dL (%)	15 ± 14	14 ± 13	15 ± 13	15 ± 15	19 ± 17	15 ± 14	10 ± 12
**Sensor glucose: Neither on**							
Mean glucose (mg/dL)	151 ± 20	157 ± 20	151 ± 19	151 ± 21	160 ± 24	149 ± 20	144 ± 16
Time in range 70 to 180mg/dL (%)	73 ± 12	66 ± 11	72 ± 11	73 ± 12	67 ± 13	74 ± 12	79 ± 10
Time <70mg/dL (%)	2.1 (1.2, 3.4)	3.3 (2.1, 4.8)	2.7 (1.8, 4.1)	2.4 (1.5, 3.4)	1.9 (1.1, 3.1)	1.9 (1.1, 3.1)	1.4 (0.7, 2.3)
Time <54mg/dL (%)	0.33 (0.15, 0.66)	0.60 (0.31, 1.09)	0.48 (0.24, 0.82)	0.41 (0.20, 0.73)	0.32 (0.16, 0.62)	0.28 (0.12, 0.57)	0.14 (0.06, 0.34)
Time >180mg/dL (%)	25 ± 12	30 ± 12	25 ± 11	25 ± 12	30 ± 14	24 ± 12	19 ± 11
Time >250mg/dL (%)	5 (2, 9)	8 (4, 12)	6 (3, 10)	6 (2, 12)	8 (4, 14)	4 (2, 8)	2 (1, 5)

* Data are presented as mean (standard deviation) or median (IQR)
