# Supplementary material for: Safety of options to ″Boost″ (enhancing insulin infusion rates) and ″Ease-off″ (reducing insulin infusion rates) in CamAPS FX Hybrid Closed-Loop system: a real-world analysis
Source: Diabetes Technol Ther. Author manuscript; Available in PMC 2025 May 24. (PMC7617696; doi:10.1089/dia.2024.0298)

Supplementary Appendix

Table S1. Sensor glucose outcomes according to duration of Boost and Ease-off.

	All users (N=7,464)	Young children aged 2-6 years (N=705)	Children aged 7 to13 years (N=991)	Teenagers aged 14 to 17 years (N=515)	Young adults aged18 to 22 years (N=421)	Adults aged 23 to65 years (N=4,590)	Older Adults aged 66 years and above (N=242)
**Total Amount of CGM data** (days)							
Boost on for 0-<3h	17,853	2,035	2,564	1,246	793	10,488	727
Boost on for 3-<6h	3,580	187	285	260	229	2,493	126
Boost on for 6-<9h	689	53	61	53	28	478	15
Boost on for 9-<12h	330	36	35	42	14	201	3
Boost on for 12-<18h	581	51	43	126	20	340	1
**Glucose at start of Boost** Mean ± SD							
Boost on for 0-<3h	229 ± 50	246 ± 50	246 ± 51	240 ± 56	246 ± 55	221 ± 46	209 ± 41
Boost on for 3-<6h	219 ± 50	228 ± 48	231 ± 48	241 ± 57	241 ± 59	212 ± 46	195 ± 36
Boost on for 6-<9h	210 ± 48	214 ± 40	222 ± 46	229 ± 53	244 ± 59	203 ± 45	179 ± 45
Boost on for 9-<12h	200 ± 49	212 ± 43	215 ± 55	207 ± 47	209 ± 66	194 ± 47	180 ± 42
Boost on for 12-<18h	203 ± 49	212 ± 42	226 ± 55	199 ± 33	235 ± 72	196 ± 46	157 ± 26
**Time in range (%)** Mean ± SD							
Boost on for 0-<3h	25 ± 21	16 ± 15	19 ± 17	24 ± 19	22 ± 18	28 ± 22	31 ± 23
Boost on for 3-<6h	42 ± 24	40 ± 22	41 ± 22	38 ± 22	34 ± 22	43 ± 25	42 ± 25
Boost on for 6-<9h	61 ± 25	57 ± 23	61 ± 26	61 ± 26	49 ± 25	62 ± 25	67 ± 30
Boost on for 9-<12h	68 ± 23	61 ± 22	67 ± 18	62 ± 22	64 ± 29	70 ± 23	74 ± 10
Boost on for 12-<18h	69 ± 20	62 ± 22	68 ± 18	66 ± 18	53 ± 30	71 ± 19	88 ± 4
**Mean Glucose** Mean ± SD							
Boost on for 0-<3h	231 ± 45	253 ± 43	249 ± 45	238 ± 48	241 ± 45	223 ± 42	215 ± 39
Boost on for 3-<6h	205 ± 48	205 ± 47	210 ± 47	216 ± 48	224 ± 53	202 ± 48	201 ± 45
Boost on for 6-<9h	171 ± 44	175 ± 42	171 ± 46	176 ± 58	192 ± 49	168 ± 41	160 ± 44
Boost on for 9-<12h	160 ± 41	169 ± 41	162 ± 45	169 ± 47	170 ± 52	156 ± 39	139 ± 28
Boost on for 12-<18h	156 ± 39	163 ± 37	162 ± 36	159 ± 32	197 ± 78	151 ± 35	133 ± 15
**Time <70 mg/dL (%)** Mean ± SD							
Boost on for 0-<3h	0.0 (0.0, 0.3)	0.0 (0.0, 0.2)	0.0 (0.0, 0.3)	0.0 (0.0, 0.6)	0.0 (0.0, 0.2)	0.0 (0.0, 0.3)	0.0 (0.0, 0.1)
Boost on for 3-<6h	0.0 (0.0, 1.3)	0.0 (0.0, 2.0)	0.0 (0.0, 1.5)	0.0 (0.0, 1.8)	0.0 (0.0, 1.5)	0.0 (0.0, 1.2)	0.0 (0.0, 0.0)
Boost on for 6-<9h	0.0 (0.0, 2.8)	0.0 (0.0, 3.4)	0.0 (0.0, 3.2)	0.0 (0.0, 3.5)	0.0 (0.0, 3.5)	0.0 (0.0, 2.3)	0.0 (0.0, 2.4)
Boost on for 9-<12h	0.3 (0.0, 3.3)	0.9 (0.0, 5.2)	1.1 (0.0, 4.4)	0.0 (0.0, 2.8)	1.4 (0.0, 4.2)	0.0 (0.0, 2.8)	0.9 (0.0, 5.8)
Boost on for 12-<18h	0.8 (0.0, 3.6)	1.7 (0.0, 3.5)	1.4 (0.0, 4.0)	1.3 (0.4, 2.3)	1.3 (0.0, 2.4)	0.3 (0.0, 3.9)	2.6 (0.0, 5.2)

**Time <54 mg/dL (%)** Mean ± SD							
Boost on for 0-<3h	0.00 (0.00, 0.00)	0.00 (0.00, 0.00)	0.00 (0.00, 0.00)	0.00 (0.00, 0.00)	0.00 (0.00, 0.00)	0.00 (0.00, 0.00)	0.00 (0.00, 0.00)
Boost on for 3-<6h	0.00 (0.00, 0.00)	0.00 (0.00, 0.00)	0.00 (0.00, 0.00)	0.00 (0.00, 0.12)	0.00 (0.00, 0.00)	0.00 (0.00, 0.00)	0.00 (0.00, 0.00)
Boost on for 6-<9h	0.00 (0.00, 0.00)	0.00 (0.00, 0.00)	0.00 (0.00, 0.00)	0.00 (0.00, 0.00)	0.00 (0.00, 0.00)	0.00 (0.00, 0.00)	0.00 (0.00, 0.00)
Boost on for 9-<12h	0.00 (0.00, 0.00)	0.00 (0.00, 1.14)	0.00 (0.00, 0.00)	0.00 (0.00, 0.00)	0.00 (0.00, 0.58)	0.00 (0.00, 0.00)	0.00 (0.00, 0.00)
Boost on for 12-<18h	0.00 (0.00, 0.34)	0.00 (0.00, 0.46)	0.00 (0.00, 0.24)	0.00 (0.00, 0.38)	0.00 (0.00, 0.00)	0.00 (0.00, 0.23)	0.35 (0.00, 0.69)
**Time >180 mg/dL (%)** Mean ± SD							
Boost on for 0-<3h	75 ± 21	84 ± 16	81 ± 18	76 ± 20	78 ± 18	71 ± 22	69 ± 23
Boost on for 3-<6h	57 ± 25	58 ± 23	58 ± 23	61 ± 22	64 ± 24	55 ± 25	57 ± 25
Boost on for 6-<9h	37 ± 26	40 ± 24	36 ± 26	36 ± 27	47 ± 26	36 ± 26	32 ± 31
Boost on for 9-<12h	30 ± 24	36 ± 22	30 ± 19	36 ± 24	33 ± 30	28 ± 24	22 ± 14
Boost on for 12-<18h	29 ± 21	36 ± 23	29 ± 19	31 ± 19	46 ± 31	26 ± 20	10 ± 7
**Time >250 mg/dL (%)** Mean ± SD							
Boost on for 0-<3h	33 (15, 56)	49 (29, 69)	49 (25, 68)	38 (17, 61)	42 (22, 60)	28 (12, 48)	20 (10, 39)
Boost on for 3-<6h	21 (7, 41)	22 (9, 40)	26 (9, 46)	28 (15, 46)	33 (17, 53)	19 (5, 38)	18 (6, 35)
Boost on for 6-<9h	6 (0, 21)	11 (1, 23)	10 (0, 23)	10 (0, 27)	25 (7, 36)	5 (0, 17)	0 (0, 4)
Boost on for 9-<12h	2 (0, 14)	8 (0, 20)	3 (0, 11)	5 (0, 21)	5 (3, 18)	1 (0, 13)	0 (0, 0)
Boost on for 12-<18h	3 (0, 13)	7 (0, 14)	5 (0, 12)	3 (0, 18)	7 (3, 40)	3 (0, 12)	0 (0, 0)
**Total Amount of CGM data** (days)							
Ease-off on for 0-<3h	11,745	1,432	1,416	523	606	7,373	395
Ease-off on for 3-<6h	3,760	304	366	166	279	2,560	85
Ease-off on for 6-<9h	1,544	166	142	50	89	1,028	69
Ease-off on for 9-<12h	746	71	67	29	41	509	29
Ease-off on for 12-<18h	324	22	35	17	59	191	1
Ease-off on for 18-24h	150	12	14	22	12	89	2
**Glucose at start of Ease-off** Mean ±SD							
Ease-off on for 0-<3h	113 ± 28	117 ± 30	112 ± 28	106 ± 29	114 ± 29	114 ± 27	109 ± 28
Ease-off on for 3-<6h	119 ± 28	121 ± 30	116 ± 26	114 ± 34	120 ± 31	120 ± 27	114 ± 22
Ease-off on for 6-<9h	123 ± 30	121 ± 33	117 ± 24	118 ± 33	125 ± 29	125 ± 30	120 ± 24
Ease-off on for 9-<12h	128 ± 32	123 ± 30	120 ± 23	120 ± 45	131 ± 25	130 ± 33	117 ± 22
Ease-off on for 12-<18h	128 ± 34	126 ± 25	121 ± 27	118 ± 48	127 ± 28	131 ± 35	114 ± 14
Ease-off on for 18-24h	131 ± 35	127 ± 25	118 ± 28	127 ± 39	143 ± 52	133 ± 35	142 ± 21
**Time in range (%)** Mean ± SD							
Ease-off on for 0-<3h	76 ± 13	74 ± 13	75 ± 12	73 ± 14	73 ± 14	77 ± 13	80 ± 13

Ease-off on for 3-<6h	71 ± 18	74 ± 16	71 ± 18	70 ± 17	66 ± 18	71 ± 18	79 ± 17
Ease-off on for 6-<9h	68 ± 21	70 ± 20	68 ± 23	68 ± 22	63 ± 22	67 ± 21	79 ± 19
Ease-off on for 9-<12h	65 ± 22	70 ± 21	70 ± 20	67 ± 25	62 ± 23	63 ± 22	75 ± 15
Ease-off on for 12-<18h	64 ± 23	71 ± 16	70 ± 18	57 ± 26	60 ± 21	64 ± 24	86 ± 20
Ease-off on for 18-24h	63 ± 22	66 ± 18	75 ± 15	69 ± 24	54 ± 24	61 ± 23	65 ± 11
**Mean Glucose** Mean ± SD							
Ease-off on for 0-<3h	121 ± 24	119 ± 24	122 ± 24	118 ± 25	126 ± 27	122 ± 24	117 ± 24
Ease-off on for 3-<6h	146 ± 29	139 ± 26	145 ± 30	146 ± 30	153 ± 32	146 ± 29	140 ± 25
Ease-off on for 6-<9h	157 ± 33	146 ± 30	155 ± 39	155 ± 37	166 ± 38	158 ± 31	149 ± 21
Ease-off on for 9-<12h	162 ± 34	149 ± 34	154 ± 35	158 ± 43	168 ± 34	165 ± 33	155 ± 21
Ease-off on for 12-<18h	165 ± 36	154 ± 27	153 ± 29	173 ± 49	177 ± 35	167 ± 36	140 ± 9
Ease-off on for 18-24h	164 ± 34	152 ± 23	148 ± 25	154 ± 43	182 ± 37	169 ± 36	157 ± 8
**Time <70 mg/dL (%)** Mean ± SD							
Ease-off on for 0-<3h	8.3 (3.5, 16.4)	10.8 (5.5, 18.4)	8.9 (4.0, 17.2)	12.3 (5.3, 22.8)	7.9 (3.5, 16.0)	7.7 (3.2, 15.2)	7.2 (3.0, 15.4)
Ease-off on for 3-<6h	2.4 (0.0, 6.7)	3.7 (0.4, 9.2)	3.3 (0.7, 7.2)	3.4 (0.1, 8.3)	2.6 (0.3, 6.0)	2.1 (0.0, 6.1)	1.8 (0.0, 5.2)
Ease-off on for 6-<9h	0.6 (0.0, 3.7)	1.8 (0.0, 6.2)	1.9 (0.0, 4.5)	0.4 (0.0, 4.7)	0.5 (0.0, 3.4)	0.3 (0.0, 2.9)	0.0 (0.0, 1.4)
Ease-off on for 9-<12h	0.4 (0.0, 2.6)	1.7 (0.0, 4.7)	0.8 (0.0, 3.3)	1.3 (0.0, 7.3)	0.8 (0.0, 2.7)	0.0 (0.0, 1.9)	1.2 (0.0, 5.0)
Ease-off on for 12-<18h	0.4 (0.0, 2.7)	0.4 (0.0, 4.5)	2.5 (0.0, 4.3)	0.0 (0.0, 5.3)	0.5 (0.0, 2.0)	0.2 (0.0, 2.4)	2.8 (0.0, 5.6)
Ease-off on for 18-24h	0.9 (0.0, 3.2)	1.7 (0.9, 4.9)	3.0 (1.3, 4.4)	1.4 (0.0, 5.6)	0.0 (0.0, 0.5)	0.3 (0.0, 2.2)	1.8 (0.0, 12.1)
**Time <54 mg/dL (%)** Mean ± SD							
Ease-off on for 0-<3h	1.15 (0.00, 3.57)	1.95 (0.52, 4.51)	1.57 (0.00, 3.89)	2.62 (0.39, 6.24)	1.18 (0.00, 3.84)	0.91 (0.00, 3.02)	0.79 (0.00, 2.65)
Ease-off on for 3-<6h	0.00 (0.00, 0.93)	0.00 (0.00, 1.70)	0.00 (0.00, 1.37)	0.00 (0.00, 1.49)	0.00 (0.00, 0.71)	0.00 (0.00, 0.73)	0.00 (0.00, 0.84)
Ease-off on for 6-<9h	0.00 (0.00, 0.00)	0.00 (0.00, 1.01)	0.00 (0.00, 0.15)	0.00 (0.00, 0.00)	0.00 (0.00, 0.00)	0.00 (0.00, 0.00)	0.00 (0.00, 0.00)
Ease-off on for 9-<12h	0.00 (0.00, 0.00)	0.00 (0.00, 0.31)	0.00 (0.00, 0.27)	0.00 (0.00, 0.60)	0.00 (0.00, 0.00)	0.00 (0.00, 0.00)	0.00 (0.00, 0.44)
Ease-off on for 12-<18h	0.00 (0.00, 0.13)	0.00 (0.00, 0.34)	0.04 (0.00, 1.69)	0.00 (0.00, 0.26)	0.00 (0.00, 0.00)	0.00 (0.00, 0.00)	0.00 (0.00, 0.00)
Ease-off on for 18-24h	0.00 (0.00, 0.23)	0.43 (0.00, 0.95)	0.00 (0.00, 0.23)	0.62 (0.00, 1.36)	0.00 (0.00, 0.00)	0.00 (0.00, 0.09)	0.00 (0.00, 0.00)
**Time >180 mg/dL (%)** Mean ± SD							
Ease-off on for 0-<3h	12 ± 12	12 ± 12	13 ± 12	12 ± 12	15 ± 14	12 ± 12	9 ± 11
Ease-off on for 3-<6h	24 ± 18	20 ± 16	24 ± 18	25 ± 18	29 ± 19	24 ± 19	17 ± 18
Ease-off on for 6-<9h	29 ± 22	25 ± 20	28 ± 24	29 ± 23	34 ± 23	30 ± 21	19 ± 19
Ease-off on for 9-<12h	33 ± 23	26 ± 22	27 ± 20	29 ± 26	36 ± 24	35 ± 23	22 ± 16
Ease-off on for 12-<18h	34 ± 24	27 ± 16	26 ± 19	40 ± 27	39 ± 22	35 ± 25	11 ± 16
Ease-off on for 18-24h	34 ± 22	30 ± 18	21 ± 16	27 ± 26	46 ± 24	37 ± 23	31 ± 4
**Time >250 mg/dL (%)** Mean ± SD							
Ease-off on for 0-<3h	0 (0, 3)	1 (0, 3)	1 (0, 4)	0 (0, 3)	1 (0, 5)	0 (0, 2)	0 (0, 1)

Ease-off on for 3-<6h	1 (0, 8)	1 (0, 6)	2 (0, 10)	2 (0, 9)	5 (0, 10)	1 (0, 7)	0 (0, 3)
Ease-off on for 6-<9h	1 (0, 10)	1 (0, 7)	4 (0, 12)	2 (0, 12)	3 (0, 16)	1 (0, 10)	0 (0, 1)
Ease-off on for 9-<12h	4 (0, 13)	1 (0, 11)	2 (0, 12)	1 (0, 12)	7 (0, 19)	4 (0, 13)	1 (0, 11)
Ease-off on for 12-<18h	6 (0, 16)	0 (0, 6)	1 (0, 13)	12 (0, 23)	15 (3, 21)	6 (0, 16)	4 (0, 8)
Ease-off on for 18-24h	8 (2, 15)	4 (2, 6)	5 (0, 15)	6 (0, 26)	17 (1, 29)	9 (3, 15)	9 (2, 9)

Table S2. Total duration of Boost and Ease-off use by month[a]

Month	Boost (Hours/30 days)		Ease Off (Hours/30 days)	
	N	Median (IQR)	N	Median (IQR)
1	5,637	11.7 (0.0, 34.6)	5,637	6.9 (0.0, 25.9)
2	7,281	10.6 (0.0, 33.5)	7,281	5.3 (0.0, 24.6)
3	5,478	11.0 (0.0, 34.8)	5,478	6.7 (0.0, 27.4)
4	5,925	10.9 (0.0, 33.3)	5,925	6.1 (0.0, 25.0)
5	5,771	10.9 (0.0, 34.4)	5,771	6.7 (0.0, 25.9)
6	5,933	10.9 (0.0, 33.1)	5,933	6.3 (0.0, 25.8)

a- Minimum of 1 day of CGM data per month to be included.

Table S3 Sensor glucose outcomes during Boost, Ease-off periods and overall using real-world data.

	**All users**	**Young children Age 1 – 6 years**	**Pre-pubertal children Age 7 – 13 years**	**Teenagers Age 14 – 17 years**	**Young adults Age 18 – 22 years**	**Adults Age 23 – 65 years**	**Older Adults Age 66 years and older**
**Number of participants**	7464	705	991	515	421	4590	242
**Age** (years)	32 ± 19	5 ± 1	10 ± 2	15 ± 1	20 ± 1	42 ± 11	71 ± 4
**Amount of CGM data per participant** (days)	89 (58, 119)	91 (58, 124)	90 (58, 124)	80 (54, 109)	77 (49, 106)	90 (61, 120)	95 (59, 122)
**CGM and closed-loop usage**							
Time using CGM and closed-loop (%)	97 (93, 99)	97 (94, 100)	96 (92, 99)	95 (89, 99)	95 (90, 99)	97 (93, 100)	98 (95, 100)
**Boost usage**							
Mean duration of Boost (min)	65 (43, 98)	47 (31, 68)	51 (32, 75)	71 (48, 101)	77 (51, 112)	71 (49, 105)	62 (40, 85)
Frequency of Boost use (per week)	4.3 (1.8, 8.6)	6.2 (3.1, 11.5)	5.3 (2.4, 9.4)	4.4 (1.9, 9.3)	3.6 (1.4, 8.0)	3.8 (1.6, 7.8)	4.4 (2.0, 8.6)
**Ease-off usage**							
Mean duration of Ease-off (min)	82 (56, 120)	64 (42, 99)	73 (50, 114)	84 (52, 130)	98 (62, 143)	85 (60, 125)	74 (53, 101)
Frequency of Ease-off use (per week)	2.4 (1.0, 5.4)	3.2 (1.4, 8.5)	2.2 (1.0, 5.1)	1.9 (0.9, 4.2)	2.7 (1.0, 5.6)	2.4 (1.0, 5.3)	2.4 (1.2, 5.0)
**Sensor glucose: Boost on**							
Glucose at start of Boost	229 ± 51	245 ± 51	246 ± 51	242 ± 58	246 ± 56	221 ± 47	209 ± 41
Mean glucose (mg/dL)	228 ± 46	248 ± 46	245 ± 47	234 ± 49	238 ± 46	220 ± 44	214 ± 39
Time in range (%)	28 ± 22	19 ± 18	21 ± 19	26 ± 20	25 ± 19	31 ± 23	31 ± 23
Time <70mg/dL (%)	0.0 (0.0, 0.5)	0.0 (0.0, 0.4)	0.0 (0.0, 0.4)	0.1 (0.0, 0.8)	0.0 (0.0, 0.5)	0.0 (0.0, 0.4)	0.0 (0.0, 0.1)
Time <54mg/dL (%)	0.00 (0.00, 0.00)	0.00 (0.00, 0.00)	0.00 (0.00, 0.00)	0.00 (0.00, 0.10)	0.00 (0.00, 0.00)	0.00 (0.00, 0.00)	0.00 (0.00, 0.00)
Time >180mg/dL (%)	72 ± 22	81 ± 18	79 ± 19	73 ± 21	75 ± 20	69 ± 23	68 ± 23
Time >250mg/dL (%)	31 (14, 54)	47 (27, 68)	46 (24, 67)	36 (18, 59)	41 (22, 60)	26 (12, 46)	20 (9, 38)
**Sensor glucose: Ease-off on**							
Glucose at start of Ease-off	114 ± 29	118 ± 32	113 ± 28	108 ± 33	115 ± 31	114 ± 28	109 ± 28
Mean glucose (mg/dL)	127 ± 27	124 ± 25	127 ± 26	125 ± 29	134 ± 32	127 ± 27	120 ± 25
Time in range 70 to 180mg/dL (%)	75 ± 14	74 ± 13	74 ± 13	71 ± 14	71 ± 15	75 ± 14	80 ± 12
Time <70mg/dL (%)	7.0 (2.9, 14.4)	8.9 (4.4, 16.7)	7.5 (3.4, 15.9)	9.9 (3.6, 18.8)	6.1 (2.5, 12.9)	6.5 (2.5, 13.1)	6.6 (2.7, 14.7)
Time <54mg/dL (%)	0.94 (0.00, 3.03)	1.74 (0.44, 4.04)	1.27 (0.17, 3.44)	1.84 (0.24, 5.44)	0.85 (0.00, 2.82)	0.76 (0.00, 2.57)	0.74 (0.00, 2.18)
Time >180mg/dL (%)	15 ± 14	14 ± 13	15 ± 13	15 ± 15	19 ± 17	15 ± 14	10 ± 12
**Sensor glucose: Overall**							
Mean glucose (mg/dL)	151 ± 20	159 ± 20	152 ± 18	152 ± 19	159 ± 23	150 ± 19	145 ± 16
Time in range 70 to 180mg/dL (%)	72 ± 11	65 ± 11	71 ± 10	72 ± 11	67 ± 12	73 ± 11	78 ± 10

Time <70mg/dL (%)	2.2 (1.3, 3.5)	3.4 (2.1, 4.9)	2.9 (1.8, 4.2)	2.5 (1.6, 3.6)	2.0 (1.3, 3.2)	2.0 (1.1, 3.2)	1.4 (0.8, 2.3)
Time <54mg/dL (%)	0.35 (0.16, 0.69)	0.60 (0.33, 1.11)	0.49 (0.26, 0.84)	0.43 (0.22, 0.78)	0.36 (0.18, 0.67)	0.29 (0.14, 0.58)	0.15 (0.08, 0.35)
Time >180mg/dL (%)	25 ± 12	31 ± 11	26 ± 11	25 ± 11	30 ± 13	24 ± 12	20 ± 10
Time >250mg/dL (%)	5 (3, 10)	9 (5, 13)	7 (4, 11)	6 (3, 11)	8 (4, 15)	5 (2, 8)	3 (1, 5)

*Data are presented as mean (standard deviation) or median (IQR)

Figure S1. Frequency of Boost and Ease-off use over the 24 hour period by age (<18 years and ≥18 years).

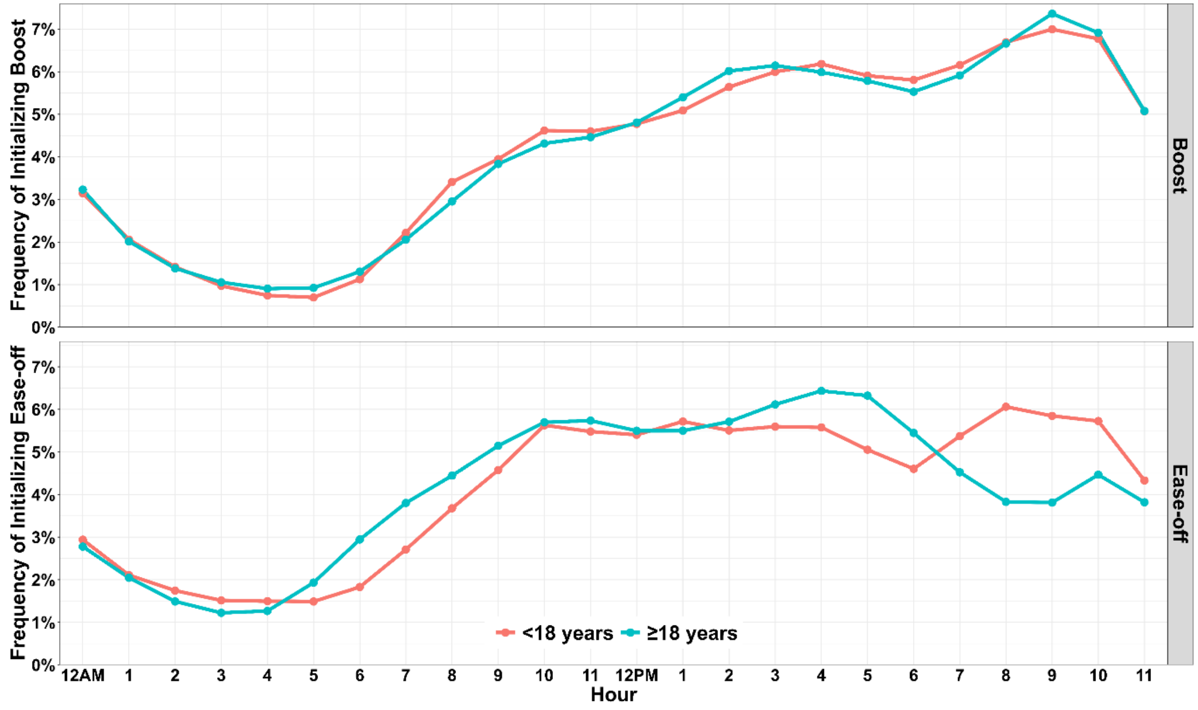

Supplement: Supplementary Appendix [file EMS205676-supplement-Supplementary_Appendix.pdf]
